# Knowledge and occupational hazards of barbers in the transmission of hepatitis B and C was low in Kumasi, Ghana

**DOI:** 10.11604/pamj.2015.20.260.4138

**Published:** 2015-03-18

**Authors:** Mohamed Mutocheluh, Kwaku Kwarteng

**Affiliations:** 1 Department of Clinical Microbiology, School of Medical Sciences, Kwame Nkrumah University of Science and Technology, Kumasi, Ghana; 2 Komfo Anokye Teaching Hospital, Kumasi, Ghana

**Keywords:** Awareness, barbers, viral hepatitis, razor blade, hair trimmers

## Abstract

**Introduction:**

Blood borne viral hepatitis transmission still ranges between 4-20% in many Ghanaian communities. Hepatocellular carcinoma (HCC) also called liver cancer is reported as the leading cause of cancer mortality among males in Ghana. We studied the knowledge and risk factors associated with barbers’ occupation in the transmission of hepatitis B virus (HBV) and hepatitis C virus (HCV).

**Methods:**

A randomized cross-sectional survey of 200 barbershops was conducted in Kumasi between January and August 2013. Barbershops, which operated continuously for more than 8 months, were selected for the study. Structured questionnaires were administered to the study participants. Data was entered and analysed in Microsoft Excel spread sheet and SPSS v12. The percentage value of each question was calculated.

**Results:**

All the barbers involved in this study used a new razor blade on every client and claimed to sterilize the hair trimmers after use on every client. The methods of sterilization; 46.5% of the barbers used the ultraviolet radiation sterilizer cabinet, 29% used 70% alcohol and 23% used antiseptic solutions. More than thirty-six percent (36.5%) and 5% of the barbers had heard of HBV and HCV respectively. Only 7% and none knew the route of transmission of HBV and HCV respectively, whereas 7% knew sharing razor blade or hair trimmer could transmit both HBV and HCV. More so, 2% knew HBV and HCV could cause cancer and 2% had received the HBV vaccine. The majority of barbers (63%) had education up to the junior secondary school level. None of the barbers used a new apron nor washed their hands after work on each client.

**Conclusion:**

Awareness of barbers about HBV or HCV and job-related factors contributing to spread of infections was very poor among the vast majority of the barbers studied. Thus, giving training for the barbers is required toward prevention of blood- borne infections associated to their profession.

## Introduction

Viral hepatitis is ten times more common than human immune deficiency virus (HIV) infection [1]. But there is no comprehensive global viral hepatitis prevention and control programme analogous to that of HIV/AIDS that provides public awareness of the disease and access to treatment of the vast numbers of people with chronic hepatitis B infection living in the poorer countries. The World Health Organization (WHO) estimates there are more than 300 million HBV, and more than 150 million HCV chronically infected individuals worldwide [2]. Studies on Ghanaian blood donors show HBV prevalence at >10%, increasing in the rural and deprived areas [3, 4], while that of HCV was estimated at more than 3% in some rural communities [4–6]. Individuals with HBV and or HCV are at risk of developing HCC. HCC is reported as the leading cause of cancer mortality among males in Ghana [7]. HBV vaccination of Ghanaian children started in 2002 [8], but the vast majority of the Ghanaian adult population have not been vaccinated, and there is no vaccine for HCV to date. The main sources of transmission of viral hepatitis are the disregard for safe blood transfusion practices and shaving from barbershops, the beauty saloons and use of unsterilized surgical instruments [9]. Razor sharing and shaving at barbershops have been identified as an important risk for blood-borne viral spread in some investigations carried out around the world [10–12]. In many parts of Africa and Asia, the widespread cultural practice of shaving at barbershops or from a roadside barber is an underestimated route of blood-borne viral disease transmission [8]. More so, razor sharing and shaving from barbershops have been identified as a risk factor for HBV spread in Italy [13] and for HCV among psychiatric patients in Egypt [14]. The 31^st^ October 2011 edition of the Science Daily newspaper, reported the need for studies to be conducted on the risk of hepatitis transmission through non-single use instruments such as hair trimmers, nail files, scissors, razor blades etc in response to a report presented to the American College of Gastroenterology's 76th Annual Scientific meeting in Washington, DC. HBV is known to survive outside the human body for seven days or more on solid surfaces such as tabletops and instruments [15–17]. In Ghana the majority of barbers operate in the informal sector of the economy and the vast majority of these barbers are either illiterate or semiliterate and with or without education beyond the senior secondary school level. Data on the knowledge or awareness of the spread of blood-borne pathogens as a result of barbers’ occupational hazards are lacking in Ghana. In this study, the awareness and risk factors associated with barbers’ occupational hazards in the transmission of HBV and HCV were evaluated.

## Methods

### Study setting

A randomized cross-sectional survey of 200 barbershops within the Kumasi metropolis was conducted between January and August 2013. The selected barbershops were drawn from both low and high socioeconomic suburbs of the Kumasi metropolis. Kumasi is the capital of the Ashanti region with estimated population of approximately 2 million people according to the 2010 population and housing census report.

### Data collection

Volunteers including staff, medical and biomedical students of the Kwame Nkrumah University of Science and Technology in Kumasi administered a self-designed structured questionnaire. The purpose of the study was explained and informed verbal consent was obtained from the study participants. The participants were assured that their identity and information would be kept confidential. Barbershops, which have been in existence and operated continuously for at least 8 months or more, were eligible for inclusion in the study. The questionnaire was interpreted in the local language (Twi) for barbers who did not understand English. The questionnaire was sectioned as follows: the first section included the demographics. The second section included questions regarding their practice, razor blade or hair trimmer change on every customer, razor blade disposal, mode of sterilization of hair trimmer or razor blade; whether antiseptic solution, ultraviolet radiation, 70% alcohol or a combination of the three are used to sterilize the instruments in question, changing of apron, powder brush and hand washing for customers. The last section included questions to assess their knowledge about HBV and HCV.

### Data analysis

Data was entered and analysed in Microsoft Excel spread sheet and SPSS v12. The percentage value of each question was calculated.

## Results

**Figure 1:**
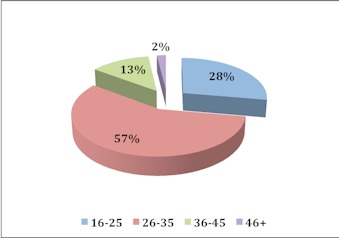
Age of barbers

**Figure 2:**
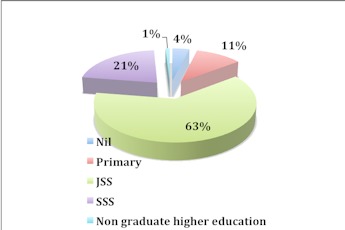
Educational background of barbers

**Figure 3:**
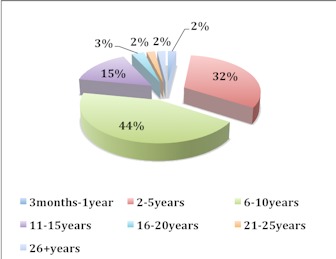
Work experience of barbers

Figure 1 shows the demographics of the study participants. All the 200 barbershops selected for the study actually participated in the study. The 100% response rate from the study participants was achieved through the kind cooperation of all the study participants. Out of the 200 barbers interviewed, 28% were aged 16-25 years, 57% were aged 26-35 years, 13% were aged 36-45 years and 2% were aged above 46 years (Figure 1). Figure 2 represents the educational background of the study participants. Individuals who completed the junior secondary school level of education were considered semiliterate or had weak educational background. Majority of the barbers were semiliterate or had weak educational background. The report shows that 63% of the study participants were junior secondary school graduates, 21% were senior secondary school graduates, 11% were primary school leavers, 4% were illiterates or never been to school before and 1% were non graduate higher education leavers. Non graduate higher education leavers were individuals who attained polytechnic, technical or vocational levels of education. Figure 3 shows the proportion of barbers categorised according to work experience. The vast majority of the barbers (76%) had a working experience ranging from 2-10 years.

**Figure 4:**
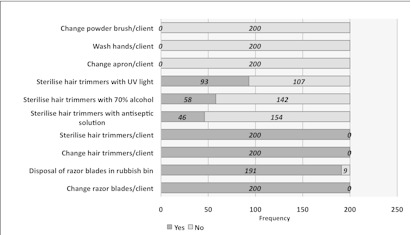
Common practises of the barbershops

Figure 4 demonstrates the daily activities of the barbers while at work. All the barbers changed the razor blade per client i.e. used new razor blade on every customer. Majority of the barbers (95.5%) disposed of the used razor blades into the bins and the remaining 4.5% buried the used razor blades. All the barbers were unable to dispose of the used hair trimmers for each client but sterilised them because they were non-disposable hair trimmers (Figure 4). The mode of sterilization of the hair trimmers was; 46.5% for barbers using ultra violet (UV) radiation, 29% used 70% alcohol, and 23% used antiseptic solution. None (0%) of the barbers changed aprons, washed hands nor changed the powder brush per client (Figure 4). Table 1 shows barber's knowledge or awareness about HBV and HCV and factors associated with their work that contributes to the spread of infections. The results showed that whereas 18% of them had come across advertisement about viral hepatitis, 36.5% had heard of HBV, 5% had heard of HCV and only 7% knew the route of transmission of HBV and none knew the transmission of HCV. More over, only 2% knew that HBV infected individuals could remain asymptomatic for several years and 7% knew HBV could be transmitted by sharing razor blades or hair trimmers but none knew the route of HCV transmission. Also, 2% of the barbers thought HBV and HCV could cause cancer while 2.5% thought HBV and HCV infections were major diseases in Ghana. When asked if the main cause of viral hepatitis was due to unawareness and illiteracy, 60% of the barbers answered the affirmative. However, only 2% of the barbers knew HBV vaccine was available in Ghana and 2% of them had received the HBV vaccine.

**Table 1:** Knowledge of barbers about HBV and HCV and job-related factors that contribute to the spread of infections (n = 200)

Items	Number	%
Come across advertisement about viral hepatitis	36	18
Heard of HBV before	73	36.5
Heard of HCV before	10	5.0
Know the route of transmission of HBV	14	7.0
Know the route of transmission of HCV	0	0.0
Know HBV positive patients remain asymptomatic	4	2.0
Know HBV and HCV can be transmitted by sharing razor blade / hair trimmer	14	7.0
Think HBV or HCV infection can cause cancer	4	2.0
Think HBV and HCV infections are major diseases in Ghana	5	2.5
Think the main cause of viral hepatitis infections is unawareness and illiteracy	120	60.0
Know HBV infections can be prevented by vaccination	4	2.0
Know HCV infections can be prevented by vaccination	0	0.0
Have take the HBV vaccine	4	2.0

## Discussion

This survey was conducted in Kumasi, Ghana to assess barbers’ knowledge about HBV and HCV and their job related practices that could be contributing to the spread of infections. The vast majority of the barbers displayed lack of knowledge about the existence of viral hepatitis caused by HBV and HCV but 97% (194/200) knew that the major risk factor in sharing used razor blades was the transmission of HIV. This means majority of barbers in Ghana are aware that HIV was transmissible via sharing of the barbers’ instruments which, is consistent with a study conducted in north West Ethiopia that showed all barbers interviewed knew that HIV was one of the biological hazards of their work [18]. Reports from many countries showed that HBV can be transferred by blade sharing and barber-related instruments [9, 11, 19–26]. All the barbers in this study did not reuse the razor blade after it was used for a client, this is in contrast with studies in other parts of the world where some barbers washed the razor blade after it was used for a client and then used again for multiple clients [11, 27]. The reasons all barbers in this study did not reuse razor blades could be attributed to the fact that razor blades are very cheap and readily available in Ghanaian cities. Moreover, Ghanaian city dwellers that visit the barbershops generally demand new razor blades to be used on them because of the awareness of risk of infection and high standard of personal hygienic practices. This study also revealed that more than 95% of the barbers disposed of their used razor blades directly into the bins, thus posing a major risk to sweepers and garbage handlers. In resource limited countries such as Ghana municipal waste dumpsites are a source of livelihood for some people who search for scraps; these scavengers are at high risk of getting infections through injuries by contaminated razor blades. It is speculated that barbers in the cities commonly use hair trimmers probably due to the availability of electricity and the influx of cheaper second hand hair trimmers. However, unlike the razor blades, the barbers could not afford to use one hair trimmer per client and were therefore compelled to reuse on multiple clients.

A UV radiation sterilizer cabinet was seen in all barbershops visited for this study but the majority of the sterilizers could only be described as storage cabinets or for display purpose only as most of the sterilizer cabinets either used mercury bulb as the UV bulb or did not have any light source in them. The reason for these flawed professional practices could be attributed to lack of information about the sterilization process. Most barbers were seen placing their hair trimmers into the sterilizer cabinets when not in use or after use on a client. But for those barbershops with heavy workload the same set of hair trimmers could be used continuously for multiple clients without sterilization. Complete sterilization of instruments and other articles placed in sterilizer cabinets cannot be achieved because the ultra violet radiation does not penetrate to all the surfaces. Also, not enough time was allowed for the radiation to completely destroy the viral DNA. More so, some microorganisms are not particularly susceptible to ultra violet radiation. It was commendable for those barbers who sterilized their hair trimmers and other instruments with 70% alcohol because the envelope proteins of HBV or HCV can be denatured or even completely destroyed. It has been reported that HBV is inactivated by 70% isopropyalcohol at 11°C for 2 minutes or a combination of beta-propriolactone and UV irradiation [16]. The challenges to the 70% alcohol mode of sterilization is that most of the barbers may over dilute the alcohol with water so they could save some money but that reduces the sterilising ability of the alcohol solution. Although the antiseptic solution is not recommended for sterilization of hair trimmers or other instruments in the barbershops, this study observed in some barbershops that the antiseptic solutions were also over diluted probably for economic gains. The antiseptic solutions are recommended for disinfection purpose only.

Generally, barbers are most likely to be exposed to blood, bodily fluids and hair flakes of their customers. Reports from the Siva region of Turkey indicate that HBV and HCV infection among barbers is extremely high compared to matched controls [28], and the fact that HBV may survive outside the body for about one week or more on table tops, solid surfaces and instruments prompted the authors to investigate how aprons, the barbers’ contaminated hands and powder brush could contribute to the transmission of HBV and HCV among people who visit the barbershops. This study also showed none of the barbershops changed their aprons, powder brushes or washed their hands after work on each client making such a professional practice a potential source of spreading blood- borne viruses such as HBV or HCV among their clients. The lack of basic knowledge of personal hygiene exhibited by the barbers is consistent with their level of education as most of them were semi illiterates. According to this study 36.5% and 5% of the barbers had heard of HBV and HCV respectively, in contrast to 39.6% reported in Pakistan for the same viruses [29]. Only 2% of the barbers in this study knew HBV and HCV could cause liver cancer in contrast with 12.5% reported in Pakistan [11]. Knowledge of the route of transmission, availability of vaccines and the main cause of HBV or HCV spread in Ghana was poor among the barbers. Also, majority of the barbers were unaware that individuals infected with HBV or HCV could remain asymptomatic for several years.

## Conclusion

In conclusion, awareness of barbers about HBV or HCV and job-related factors capable of contributing to the spread of infections was very poor for the vast majority of the barbers studied. Training workshops for barbers’ occupational hazards from the local health authorities could contribute significantly in the reduction of the spread of these blood-borne pathogens among people who visit the barbershops.
